# Parkinson’s disease: deep learning with a parameter-weighted structural connectome matrix for diagnosis and neural circuit disorder investigation

**DOI:** 10.1007/s00234-021-02648-4

**Published:** 2021-01-22

**Authors:** Koichiro Yasaka, Koji Kamagata, Takashi Ogawa, Taku Hatano, Haruka Takeshige-Amano, Kotaro Ogaki, Christina Andica, Hiroyuki Akai, Akira Kunimatsu, Wataru Uchida, Nobutaka Hattori, Shigeki Aoki, Osamu Abe

**Affiliations:** 1 Department of Radiology, The Institute of Medical Science, The University of Tokyo, 4-6-1 Shirokanedai, Minato-ku, Tokyo, 108-8639 Japan; 2 Department of Radiology, Juntendo University Graduate School of Medicine, 2-1-1 Hongo, Bunkyo-ku, Tokyo, 113-8421 Japan; 3 Department of Neurology, Juntendo University Graduate School of Medicine, 2-1-1 Hongo, Bunkyo-ku, Tokyo, 113-8421 Japan; 4 Department of Radiological Sciences, Graduate School of Human Health Sciences, Tokyo Metropolitan University, 7-2-10 Higashi-Ogu, Arakawa-ku, 116-8551 Tokyo, Japan; 5 Department of Radiology, Graduate School of Medicine, The University of Tokyo, 7-3-1 Hongo, Bunkyo-ku, Tokyo, 113-8655 Japan

**Keywords:** Parkinson disease, Connectome, Magnetic resonance imaging, Deep learning, Artificial intelligence

## Abstract

**Purpose:**

To investigate whether Parkinson’s disease (PD) can be differentiated from healthy controls and to identify neural circuit disorders in PD by applying a deep learning technique to parameter-weighted and number of streamlines (NOS)–based structural connectome matrices calculated from diffusion-weighted MRI.

**Methods:**

In this prospective study, 115 PD patients and 115 healthy controls were enrolled. NOS-based and parameter-weighted connectome matrices were calculated from MRI images obtained with a 3-T MRI unit. With 5-fold cross-validation, diagnostic performance of convolutional neural network (CNN) models using those connectome matrices in differentiating patients with PD from healthy controls was evaluated. To identify the important brain connections for diagnosing PD, gradient-weighted class activation mapping (Grad-CAM) was applied to the trained CNN models.

**Results:**

CNN models based on some parameter-weighted structural matrices (diffusion kurtosis imaging (DKI)–weighted, neurite orientation dispersion and density imaging (NODDI)–weighted, and *g*-ratio-weighted connectome matrices) showed moderate performance (areas under the receiver operating characteristic curve (AUCs) = 0.895, 0.801, and 0.836, respectively) in discriminating PD patients from healthy controls. The DKI-weighted connectome matrix performed significantly better than the conventional NOS-based matrix (AUC = 0.761) (DeLong’s test, *p* < 0.0001). Alterations of neural connections between the basal ganglia and cerebellum were indicated by applying Grad-CAM to the NODDI- and *g*-ratio-weighted matrices.

**Conclusion:**

Patients with PD can be differentiated from healthy controls by applying the deep learning technique to the parameter-weighted connectome matrices, and neural circuit disorders including those between the basal ganglia on one side and the cerebellum on the contralateral side were visualized.

## Introduction

Parkinson’s disease (PD) is a progressive disease that is characterized by degeneration of mesencephalic dopamine neurons. The prevalence of PD is increasing. The number of patients with PD has more than doubled globally from 1990 to 2015 [1], and worldwide in 2016, 6.1 million individuals had PD [2]. Although MRI is used for the diagnosis of many neurological disorders, PD does not show abnormal findings on conventional MRI. Patients with PD show extrapyramidal symptoms such as resting tremor, bradykinesia, rigidity, and postural reflex disturbance, and diagnosis of PD is made based on the clinical time course, symptoms, and physical findings.

Advancements in diffusion MRI techniques have allowed researchers to capture imaging findings of PD [3] by using diffusion tensor imaging (DTI) [4], diffusion kurtosis imaging (DKI) [5], and neurite orientation dispersion and density imaging (NODDI) [6]. Parameters derived from *g*-ratio analysis [7] may also capture microstructural changes in PD [8]. The structural connectome matrix, or number of streamlines (NOS)-based connectome matrix, is calculated from diffusion-weighted MRI, represents neural connections of the whole brain [9], and also has potential to reveal abnormalities in neural connections in some neurological and psychological disorders [10] including PD [11]. While NOS-based matrices represent only microstructural fiber counts, elements of the connectome matrix can also be weighted using parameters derived from advanced diffusion MRI techniques. Such parameter-weighted matrices have a potential to represent tissue properties and to have higher sensitivity to capture the characteristics of neurological diseases. For example, the usefulness of the *g*-ratio-weighted connectome matrix over the NOS-based matrix was reported in the diagnosis of multiple sclerosis [12]. Use of a parameter-weighted connectome matrix from DTI, DKI, NODDI, and *g*-ratio analyses has the potential to diagnose PD better than the conventional NOS-based matrix; however, to the best of our knowledge, no investigations have used such parameter-weighted connectome matrices to evaluate PD.

Deep learning, which is an artificial intelligence approach, allows automated analyses of large-volume data such as images without explicitly teaching knowledge to computers [13]. Convolutional neural network (CNN), a deep learning strategy, has shown high performance in image recognition tasks [14]. Therefore, application of CNN to radiological imaging diagnosis has been gaining wide attention [15–17], and researchers have successfully applied this technique to several imaging diagnosis tasks with radiography [18, 19], CT [20, 21], and MRI [22, 23]. Deep learning would also have a potential to handle connectome matrix which has a large data volume comprising thousands of independent elements. One of the weaknesses of deep learning is how it makes decisions has been difficult to interpret for us [15]. However, visualization of regions of images where deep learning models focus has become possible with gradient-weighted class activation mapping (Grad-CAM) [24]. This technique may allow visualizing alterations of neural circuits in PD patients when it is applied to CNNs which is trained with connectome matrices to diagnose PD.

The aim of this study was to investigate whether patients with PD can be differentiated from healthy controls by applying a deep learning technique to a parameter-weighted connectome matrix with higher performance than a NOS-based structural connectome matrix calculated from MRI obtained with a 3-Tesla MRI unit and to find neural circuit disorders associated with PD by using Grad-CAM techniques.

## Methods

In this prospective study, which was approved by our institutional review board, MRI data of patients with PD and healthy controls were analyzed. There is subject overlap (64 out of 230) with previous studies [25, 26], which used different analyzing methods (tract-of-interest, tract-based spatial statistics, and gray matter-based spatial statistics analyses) from the current study. Those studies did not use deep learning technique or connectome matrices in the evaluations of PD. Written informed consent was obtained from each participant.

### Participants

Patients with PD and healthy controls who visited Juntendo University Hospital (blinded for the review process) between February 2017 and October 2018 were enrolled in this study. Specialized neurologists made the diagnosis of PD according to clinical diagnostic criteria for PD of the Movement Disorder Society [27]. Patients showed parkinsonism and responded to antiparkinsonian therapy. Patients with apparent signs of other diseases which shows parkinsonism were not included. Disease duration from onset to MRI examination, disease severity, and dominant side are shown in Table 1. Healthy controls had no history of neurological diseases. Participants with a brain infarction or incomplete MRI examination were excluded. Age- and gender-matched participants in the PD and healthy control groups were selected before the deep learning and main analyses.

**Table 1:** Patient demographics data

	PD	Healthy controls	Comparison (*p* value)
Number of participants	115	115	N/A
Men/women	52/63	61/54	0.291
Mean age (years)	68.9 ± 6.9	69.8 ± 3.0	0.194
Mean disease duration from onset to MRI examination (years)	10.4 ± 5.7	N/A	N/A
Median Unified Parkinson’s Disease Rating Scale-III motor subscale score	22 (13.5–32)	N/A	N/A
Hoehn-Yahr stage (1/2/3/4/5)	7/33/54/20/1	N/A	N/A
Dominant side (left/right/none)	52/60/3	N/A	N/A

Note: Data are shown as the mean ± standard deviation or median (interquartile range), where applicable. For comparisons of age and gender, the Student *t* test and chi-square test, respectively were performed

*N/A*, not applicable

### MRI data acquisition

MRI examinations were performed with a 3-T MRI unit (MAGNETOM Prisma; Siemens Healthcare) using a 64-channel head coil. Multi-shell diffusion-weighted MRI, magnetization transfer saturation images, and T1-weighted images were obtained.

Multi-shell diffusion-weighted MRIs were obtained with *b*-values of 0, 1000, and 2000 s/mm^2^ along 64 uniformly distributed directions for each shell with spin-echo echo-planar imaging. We also acquired standard and reverse phase-encoded blipped images with no diffusion weighting (blip up and blip down) to correct for the magnetic susceptibility-induced distortions related to the echo-planar imaging acquisitions [28]. The data were corrected for eddy currents, susceptibility-induced geometric distortions, and inter-volume motion using the EDDY and TOPUP toolboxes [28]. Acquisition parameters for the multi-shell diffusion-weighted MRIs were the following: repetition time (TR) of 3300 ms, echo time (TE) of 70 ms, field of view (FOV) of 229 × 229 mm, voxel size of 1.8 × 1.8 × 1.8 mm^3^, matrix of 130 × 130, 65 slices, number of excitations of 1, and acquisition time of 7.29 min.

To calculate the magnetization transfer (MT) saturation index, dual excitation three-dimensional multi-echo fast low-angle shot sequences were performed with predominant T1, proton density, and MT weighting. The excitation of MT-weighted images was preceded by an off-resonance Gaussian-shaped radiofrequency pulse under the following conditions: frequency offset from water resonance, 1.2 kHz; pulse duration, 9.984 ms; and nominal flip angle, 500°. Other acquisition parameters for MT saturation imaging were as follows: MT-off and MT-on scanning (TR/TE = 24/2.53 ms, flip angle = 5°) and T1-weighted imaging (TR/TE = 10/2.53 ms, flip angle = 13°); parallel imaging using a generalized auto-calibrating partially parallel acquisition factor in the phase-encoding direction, 2; 7/8 partial Fourier acquisition in the partition direction; bandwidth, 260 Hz/pixel; slice thickness, 1.8 mm; number of slices, 104; FOV, 224 × 224 mm, and matrix, 128 × 128. The total acquisition time was 6.25 min.

As structural data, three-dimensional T1-weighted images using magnetization-prepared 180° radiofrequency pulses and rapid gradient-echo sequences were also acquired. Acquisition parameters for the T1-weighted images were the following: TR/TE = 15/3.54 ms, inversion time = 1100 ms, voxel size of 0.86 × 0.86 × 0.86 mm^3^, and acquisition time, 5.14 min.

### Diffusion analyses (DTI, DKI, NODDI, and *g*-ratio analyses)

An ordinary least square was applied to the diffusion-weighted MRI data with *b* = 0 and 1000 s/mm^2^ to produce FA, mean diffusivity, axial diffusivity, and radial diffusivity based on standard formulae [4].

The diffusional kurtosis estimator [29] was implemented in MATLAB (Math-Works, Natick, MA, USA) to generate AK, MK, and RK maps.

The resulting diffusion-weighted MRIs were fitted to the NODDI model [6] using the NODDI Matlab Toolbox5 (http://www.nitrc.org/projects/noddi_toolbox) and Accelerated Microstructure Imaging via Convex Optimization [30]. Maps of the ICVF, orientation dispersion index, and isotropic volume fraction were generated.

Using an in-house MATLAB script, MT saturation data were analyzed to calculate the myelin volume fraction (MVF). To estimate MVF maps, a calibration factor of 0.1 was subsequently used to obtain a *g*-ratio of 0.7 in the corpus callosum as previously suggested [31]. The AVF was calculated based on parameters of the NODDI model (ICVF and isotropic volume fraction) and MT saturation (myelin volume fraction), as follows [7]:

$$ \mathrm{AVF}=\left(1-\mathrm{MVF}\right)\left(1-\mathrm{ISOVF}\right)\mathrm{ICVF} $$

where ISOVF denotes isotropic volume fraction. Finally, the *g*-ratio was calculated using the myelin volume fraction and AVF for each voxel using the following equation [7]:

$$ g-\mathrm{ratio}=\sqrt{\frac{\mathrm{AVF}}{\left(\mathrm{MVF}+\mathrm{AVF}\right)}} $$

### Connectome pre-processing and construction

By using the functional MRI of the Brain Software Library, Version 5.0.9 [32], pre-processing (registration, removing non-brain tissue from three-dimensional T1-weighted images, segmentation [estimation of partial volume fractions of white matter/cortical gray matter/deep gray matter/cerebrospinal fluid], and obtaining gray matter-white matter interface mask) was performed. Then, nodes were obtained according to Desikan-Killiany cortical atlas segmentation [33]. With the MRtrix software package (Brain Research Institute, http://www.brain.org.au/software/), whole-brain tractograms were generated using probabilistic multi-shell, multi-tissue constrained spherical deconvolution tracking from multi-shell diffusion-weighted MRI data. For fiber tracking, data with b-values of 1000 and 2000 s/mm^2^ were used, and 5 × 10^7^ streamlines were seeded from the white matter fiber orientation distribution function.

**Fig. 1:**
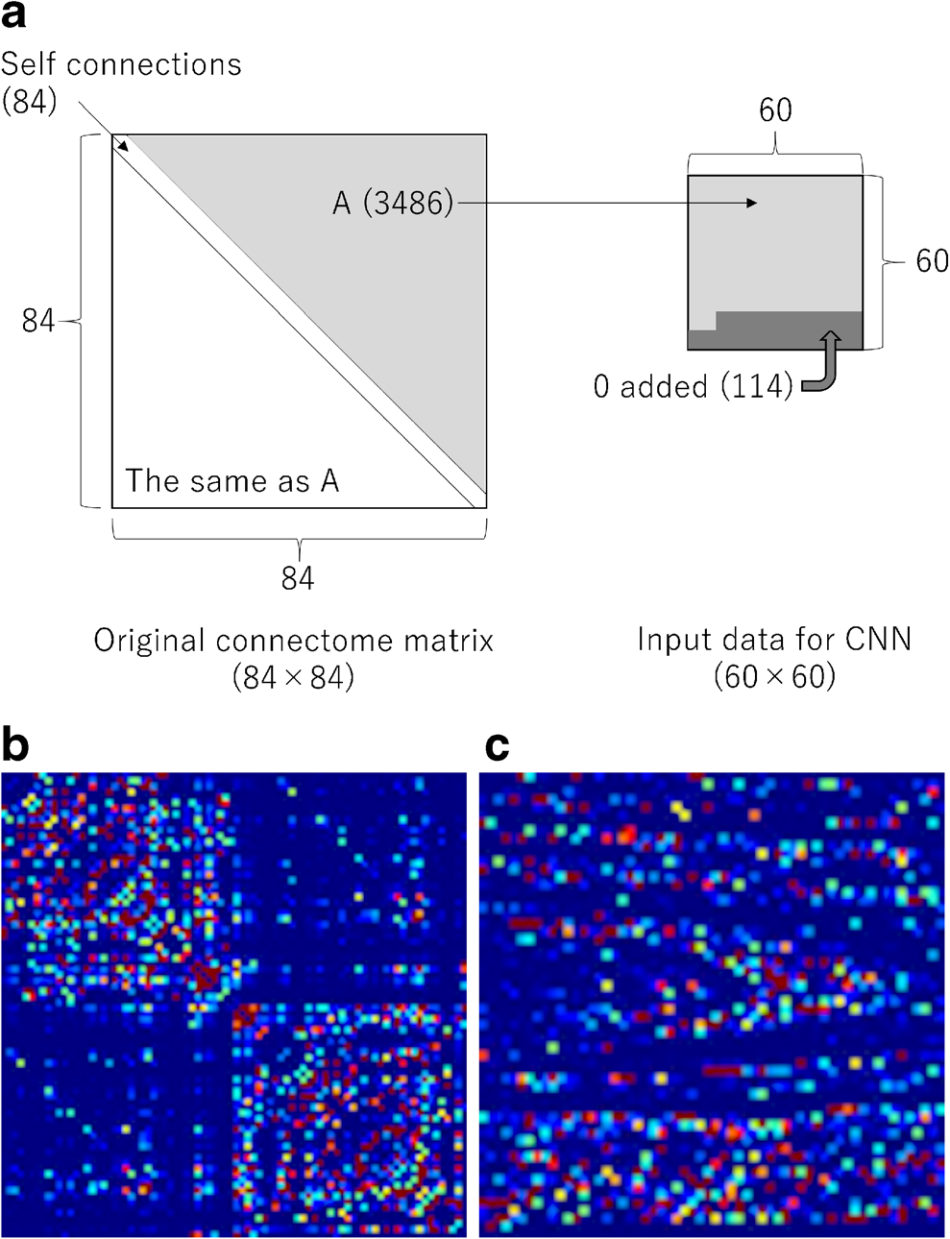
**a** NOS-based connectome matrix, which is presented as a color map, of a 68-year-old woman with PD. **b** Preparation of the input data for deep learning with the convolutional neural network (CNN). Numbers of elements are shown in parentheses. **c** Input data presented as a color map for the CNN of the same patient as in **a**. Red and blue colors indicate higher and lower values, respectively

To map the NOS-based connectome for each individual, the total NOS interconnecting each pair of regions was enumerated and stored in a connectivity matrix. Then, the NOS interconnecting each pair of nodes was enumerated, resulting in a connectome matrix with 84 × 84 components (Fig. 1a). Furthermore, DTI-, DKI-, NODDI-, and *g*-ratio-weighted connectome matrices were obtained by multiplying the NOS by the tract-averaged values of each parameter [12]. Therefore, from each patient, a total of 14 connectome matrices (one NOS-based, four DTI parameter-weighted [fractional anisotropy (FA), mean diffusivity, axial diffusivity, and radial diffusivity], three DKI parameter-weighted [mean kurtosis (MK), axial kurtosis (AK), and radial kurtosis (RK)], three NODDI parameter-weighted [intracellular volume fraction (ICVF), orientation dispersion index, isotropic volume fraction], and three *g*-ratio parameter-weighted [axon volume fraction (AVF), myelin volume fraction, and *g*-ratio] matrices) were generated.

### Training of the CNN

Deep learning was performed with a computer equipped with the graphical processing unit of Quadro P5000 (NVIDIA), 64-GB random access memory, and a Core i9-9900K (Intel). To perform deep learning, the programming language, Python 3.6.4, and the deep learning framework, Chainer 4.0.0, were used.

Input data were preprocessed. To reduce the dimension of input data, the connectome matrices were preprocessed. From the original connectome matrix (84 × 84 elements) (Fig. 1a), diagonal elements that represent self-connection were excluded (84 × 84–84 elements) (Fig. 1b). We also extracted only the independent 3486 elements ((84 × 84–84)/2 = 3486). After adding 114 zeros (3486 + 114 = 3600), the data were transformed to 60 × 60 matrix data (Fig. 1c). This 60 × 60 matrix was used as input data for deep learning with CNN. The input data were normalized by dividing by the maximum value within the matrix.

**Fig. 2:**
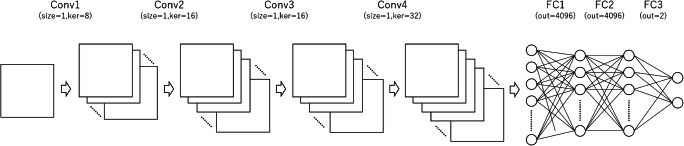
The CNN comprised four convolutional layers with channels of 8, 16, 16, and 32. Because the distance between two elements within the connectome matrix does not represent spatial distance as in general images, we customized the structure of the CNN: **a** a filter size of 1 was used for all convolutional layers and **b** max-pooling layers were omitted from the CNN. The processed data were further processed at three fully connected layers with the number of units of 4096, 4096, and 2. Conv, convolutional layer; FC, fully connected layer; ker, numbers of kernels of the convolutional layer; out, numbers of output values of the fully connected layer

As teaching data, two-element vector data (PD [0, 1] vs. healthy control [1, 0]) were used. The structure of the CNN is illustrated in Fig. 2. Errors between output data and teacher data (PD vs. healthy control) were calculated with softmax cross-entropy. The CNN was updated with a minibatch size of 15 and with an optimizer of AdaGrad [34] so that the error became small. The number of epochs for the training was 20. In the training phase, data augmentation was performed by adding Gaussian noise with a mean of 0 and a standard deviation of 0.002, 0.004, 0.006, 0.008, and 0.010 to the input data. Therefore, data were augmented 6-fold in the training phase of the CNN. The hyperparameters were determined before the main analyses by using NOS-based matrices of PD patients and healthy controls who were excluded from the study due to age and gender matching.

### Evaluating performance of the trained models

To evaluate the performance of the model, 5-fold cross-validation was performed. Data of patients with PD and healthy controls were randomly divided into five groups. Training and validation were repeated 10 times (trials) in each fold. The performance of the model that showed the best among the 10 trials was recorded for each fold. Then, by pooling the data across 5-fold, the performance of the model was evaluated.

### Grad-CAM

To visualize the regions where the developed CNN focused in diagnosing PD, the Grad-CAM technique [24] was applied to the connectome matrices of patients with PD who were diagnosed correctly in the validation. The target layer was set at the 4th convolutional layer. The data for each patient with PD were normalized and then pooled. To assess whether the relations between the dominant side and the neural circuit alteration, subanalyses were also performed by separately pooling the data for patients according to the dominant side (left or right).

### Statistics

Statistical analyses were performed with EZR version 1.37 (http://www.jichi.ac.jp/saitama-sct/SaitamaHP.files/statmedEN.html), which is a graphical user interface of R 2.4.0 (https://www.r-project.org/). Continuous and binominal data were compared with the Student *t* test and chi-square test, respectively. The performance of the CNN model for differentiating patients with PD from healthy controls was evaluated with receiver operating characteristic analyses. Areas under the receiver operating characteristic curve (AUCs) of the model that showed the best performance among each evaluation category (NOS, DTI, DKI, NODDI, *g*-ratio) were compared across categories with DeLong’s test. Because of multiple comparisons across five categories, a *p* value less than 0.005 (= 0.05/10, Bonferroni correction) was considered to indicate statistical significance. Sensitivity, specificity, and accuracy in diagnosing PD were calculated using CNN’s output value of 0.5 as a cut-off.

## Results

### Background of study participants

In this study, 115 patients with PD (mean age with standard deviation, 68.9 ± 6.9 years, 52 men and 63 women) and 115 healthy controls (mean age with standard deviation, 69.8 ± 3.0 years, 61 men and 54 women) were enrolled. Other background information is summarized in Table 1.

### Performance of models for diagnosing PD

**Fig. 3:**
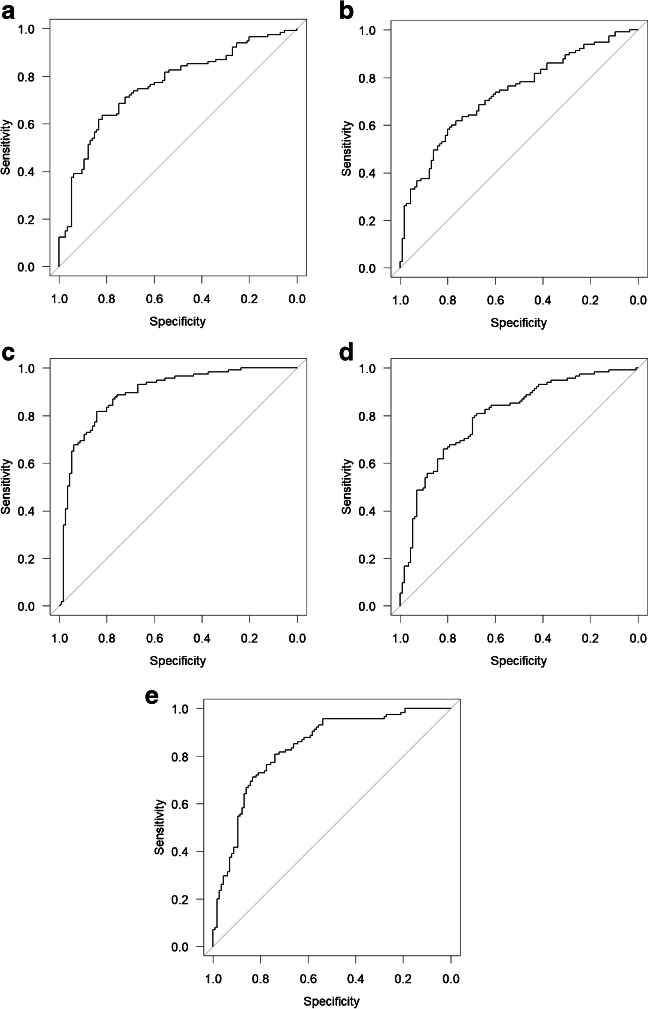
Receiver operating characteristic curves to differentiate PD from healthy controls with **a** NOS-based, **b** FA-weighted, **c** RK-weighted, **d** ICVF-weighted, and **e** AVF-weighted connectome matrices

The diagnostic performance of the trained CNN models for differentiating patients with PD from healthy controls is summarized in Table 2. AK-weighted, MK-weighted, RK-weighted, ICVF-weighted, and AVF-weighted matrices were found to show AUCs of over 0.800 in diagnosing PD (AUC = 0.891, 0.878, 0.895, 0.801, and 0.836, respectively), while NOS-based matrix showed AUC of 0.761. Among the DTI, DKI, NODDI, and *g*-ratio parameters, FA, RK, ICVF, and AVF performed the best, respectively (AUCs = 0.733, 0.895, 0.801, and 0.836, respectively) (Fig. 3). We compared the AUCs of the model that showed the best performance among each evaluation category (NOS, DTI, DKI, NODDI, *g*-ratio) (Table 3). The RK-weighted connectome matrix (best among DKI) showed significantly better performance compared with ICVF-weighted (best among NODDI), NOS-based, and FA-weighted (best among DTI) matrices (*p* = 0.0004, *p* < 0.0001, and p < 0.0001, respectively). AVF-weighted connectome matrix (best among *g*-ratio) performed significantly better than the FA-weighted connectome matrix (best among DTI) for diagnosing PD (*p* = 0.0022).

**Table 2:** Areas under the receiver operating characteristic curve for diagnosing Parkinson’s disease with the convolutional neural network

Metrics	Cross-validation					Pooled
	1	2	3	4	5	
NOS	0.688	0.767	0.764	0.887	0.843	0.761 (0.698–0.823)
DTI						
Axial diffusivity	0.747	0.722	0.713	0.664	0.864	0.715 (0.649–0.781)
Mean diffusivity	0.726	0.656	0.713	0.618	0.783	0.672 (0.602–0.741)
Radial diffusivity	0.667	0.633	0.703	0.684	0.871	0.698 (0.631–0.766)
Fractional anisotropy	0.703	0.724	0.775	0.665	0.853	0.733 (0.669–0.798)
DKI						
Axial kurtosis	0.839	0.941	0.972	0.911	0.957	0.891 (0.848–0.934)
Mean kurtosis	0.864	0.845	0.868	0.860	0.989	0.878 (0.833–0.923)
Radial kurtosis	0.847	0.879	0.913	0.854	0.987	0.895 (0.853–0.937)
NODDI						
Intracellular volume fraction	0.784	0.847	0.798	0.747	0.896	0.801 (0.744–0.858)
Isotropic volume fraction	0.688	0.798	0.745	0.730	0.868	0.749 (0.686–0.811)
Orientation dispersion	0.561	0.760	0.743	0.662	0.817	0.691 (0.623–0.759)
*g*-ratio						
Axon volume fraction	0.749	0.871	0.837	0.926	0.915	0.836 (0.784–0.888)
Myelin volume fraction	0.681	0.773	0.790	0.788	0.834	0.763 (0.701–0.824)
*g*-ratio	0.665	0.760	0.830	0.786	0.866	0.767 (0.706–0.827)

Note: The 95% confidence interval is shown in parentheses

**Table 3:** Comparisons of the pooled areas under the receiver operating characteristic curve for diagnosing Parkinson’s disease with the convolutional neural network

Metrics	AUC	Comparisons (p value)			
		AVF	ICVF	NOS	FA
RK	0.895	0.0263	0.0004*	<0.0001*	<0.0001*
AVF	0.836	N/A	0.0259	0.0295	0.0022*
ICVF	0.801	N/A	N/A	0.2740	0.0367
NOS	0.761	N/A	N/A	N/A	0.4720
FA	0.733	N/A	N/A	N/A	N/A

Note: For comparisons, DeLong’s test was performed. Because of the multiple comparisons, Bonferroni correction was applied, and *p* < 0.0050 (=0.05/10) was considered statistically significant

*AUC*, area under the receiver operating characteristic curve; *AVF*, axon volume fraction; *FA*, fractional anisotropy; *ICVF*, intracellular volume fraction; *N/A*, not applicable; *NOS*, number of streamlines; *RK*, radial kurtosis

*Statistically significant difference

Sensitivity, specificity, and accuracy of models that showed the best performance in each category are shown in Table 4.

**Table 4:** Sensitivity, specificity, and accuracy of the models (pooled across 5-fold) for diagnosing Parkinson’s disease

Metrics	Sensitivity (%)	Specificity (%)	Accuracy (%)
RK	77 (89/115)	85 (98/115)	81 (187/230)
AVF	77 (89/115)	76 (87/115)	77 (176/230)
ICVF	79 (91/115)	69 (79/115)	74 (170/230)
NOS	75 (86/115)	65 (75/115)	70 (161/230)
FA	67 (77/115)	67 (77/115)	67 (154/230)

Note: Sensitivity, specificity, and accuracy were calculated using a cut-off value of 0.5 for output from the convolutional neural network

*AVF*, axon volume fraction; *FA*, fractional anisotropy; *ICVF*, intracellular volume fraction; *NOS*, number of streamlines; *RK*, radial kurtosis

### Grad-CAM results

**Fig. 4:**
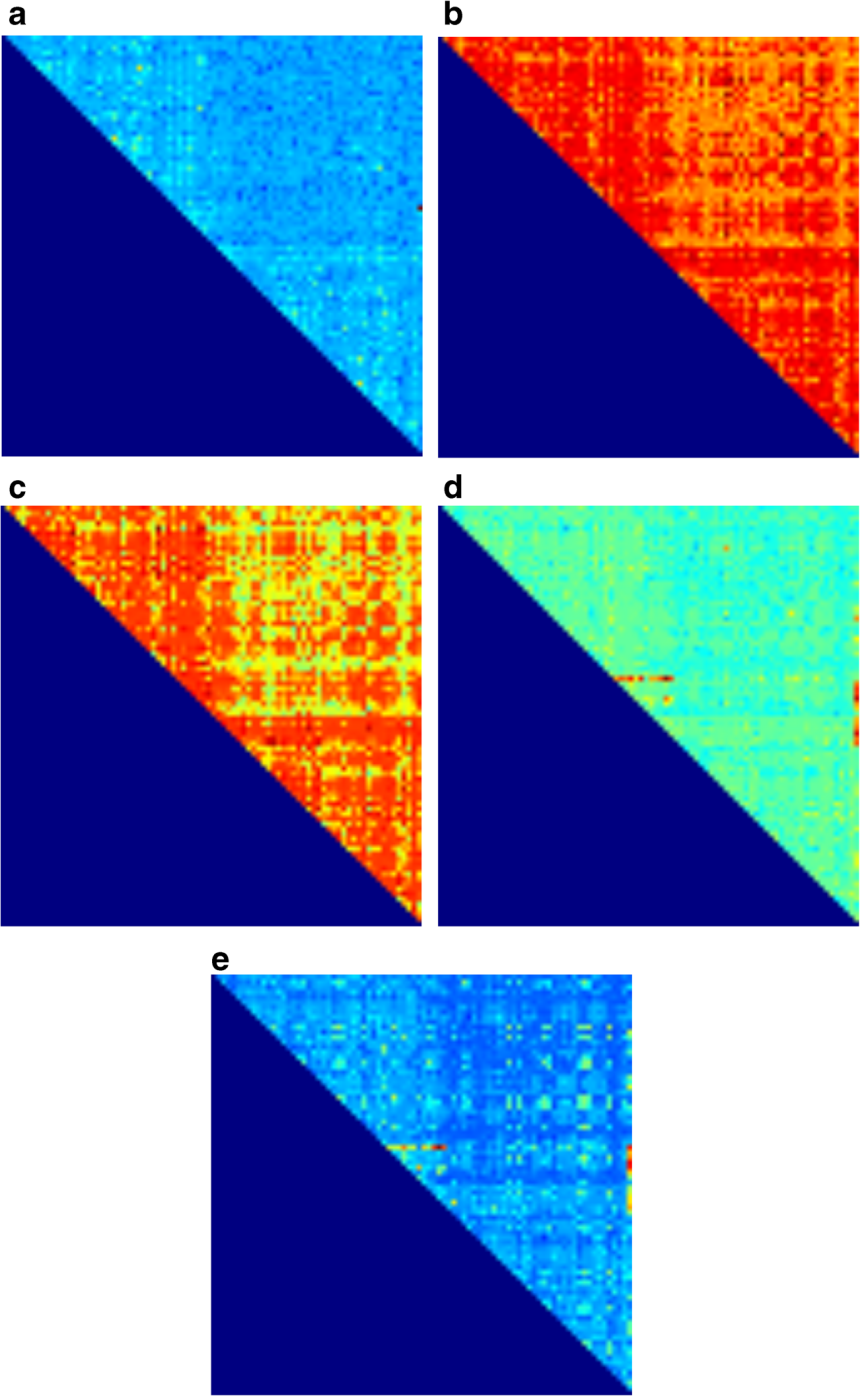
Results of Grad-CAM, which was reorganized to 84 × 84, for **a** NOS-based, **b** FA-weighted, **c** RK-weighted, **d** ICVF-weighted, and **e** AVF-weighted connectome matrices are shown. Left/top and right/bottom halves of the matrix indicate left and right brain regions, respectively. Red and blue colors indicate higher and lower values, respectively, obtained from Grad-CAM analysis

**Fig. 5:**
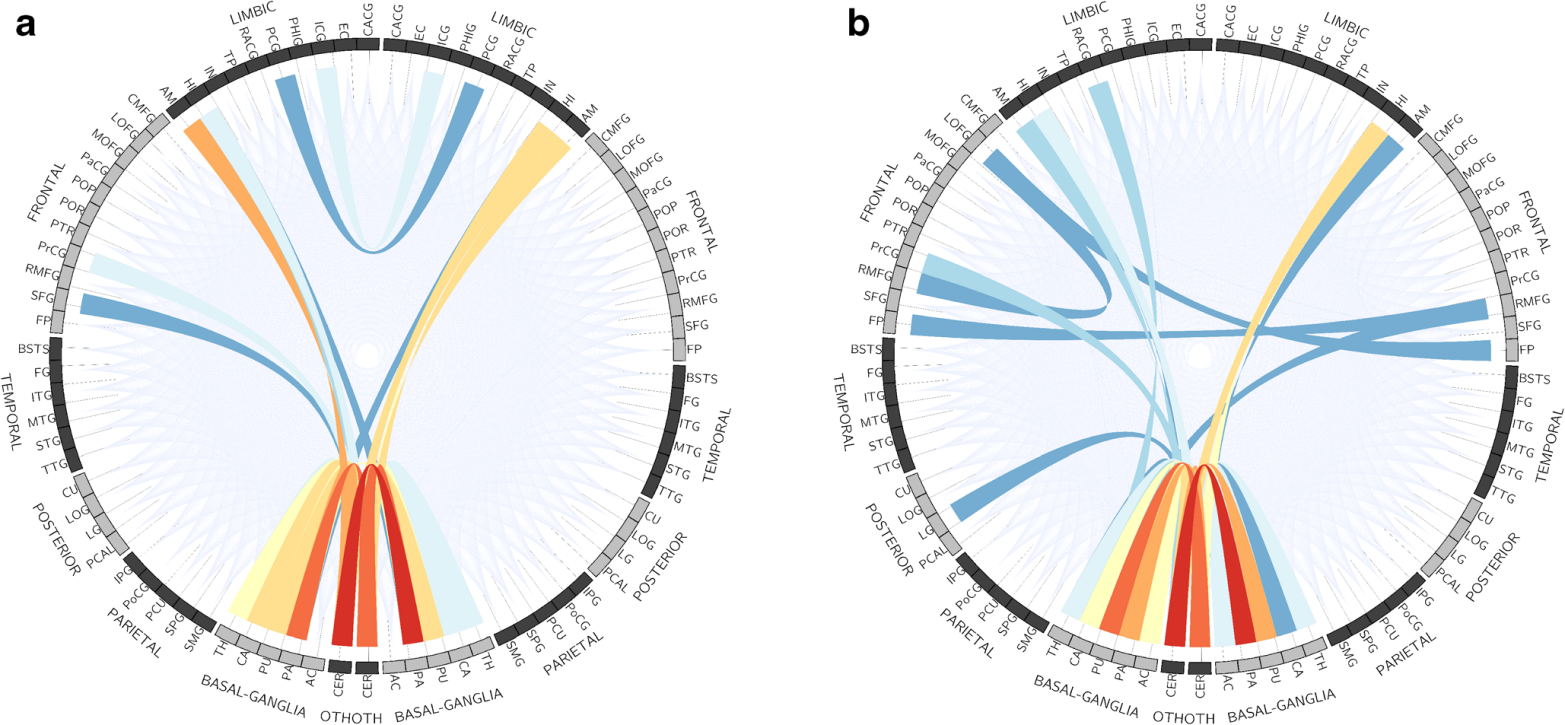
Top 30 neural connections focused on by Grad-CAM analysis for differentiating patients with PD from healthy controls with **a** ICVF-weighted and **b** AVF-weighted connectome matrices. The more intensely focused connections are represented in a more reddish color

Regions in which the trained CNN focused, which were revealed with Grad-CAM, are shown in Fig. 4. Generally, Grad-CAM result images are blurred due to the use of kernel size of more than 1 in convolutional layers and due to the use of max-pooling layers. In this study, the customized structure of our CNN (Fig. 2) allowed pin-point visualization in Grad-CAM analysis. The trained CNN model focused on many connections on the RK-weighted connectome matrix (Fig. 4c) and no specific trend in areas of focus for neural connections was found. On the contrary, ICVF-weighted and AVF-weighted connectome matrices focused on some specific neural connections, which are indicated as reddish color dots in Fig. 4d, e. These neural connections are summarized in Fig. 5 and Table 5. For these two matrices, connections between the basal ganglia and the cerebellum played important roles in discriminating patients with PD from healthy controls.

**Table 5:** Neural circuit alterations indicated by Grad-CAM analysis in AVF-weighted and ICVF-weighted matrices

Metrics	Order	Neural circuit	
AVF	1	Left cerebellar cortex	Right pallidum
	2	Left putamen	Right cerebellar cortex
	3	Left pallidum	Right cerebellar cortex
	4	Left cerebellar cortex	Right cerebellar cortex
	5	Left cerebellar cortex	Right putamen
	6	Left cerebellar cortex	Right hippocampus
	7	Left cerebellar cortex	Left pallidum
	8	Left caudate	Right cerebellar cortex
	9	Right hippocampus	Right cerebellar cortex
	10	Left pallidum	Left accumbens area
ICVF	1	Left cerebellar cortex	Right pallidum
	2	Left pallidum	Right cerebellar cortex
	3	Right pallidum	Right cerebellar cortex
	4	Left cerebellar cortex	Left pallidum
	5	Left cerebellar cortex	Left amygdala
	6	Left caudate	Right cerebellar cortex
	7	Left cerebellar cortex	Right hippocampus
	8	Left putamen	Right cerebellar cortex
	9	Left cerebellar cortex	Left caudate
	10	Left cerebellar cortex	Right putamen

*AVF*, axon volume fraction; *ICVF*, intracellular volume fraction

## Discussion

In this study, by applying the deep learning technique to DKI-, NODDI-, and *g*-ratio-weighted structural connectome matrices obtained with a 3-T MRI unit, we found that PD could be diagnosed with moderate performance. Deep learning models that were trained with the DKI-weighted connectome matrix performed significantly better than those trained with the NOS-based connectome matrix. By using the Grad-CAM technique, we found that the trained CNN focused on connections between the basal ganglia and the cerebellum for ICVF- and AVF-weighted connectome matrices, whereas no specific trend was found in the areas of focus for neural connections for the RK-weighted connectome matrix for differentiating patients with PD from healthy controls.

Parameter-weighted connectome matrix has been gaining attention recently in the evaluations of neurological diseases [12]. While NOS-based connectome matrix represents only the strength of the connectivity between brain regions, the parameter-weighted matrix can represent other tissue properties. In the current study, DKI parameters-weighted, ICVF-weighted, and AVF-weighted matrices were found to show AUCs of over 0.800 in diagnosing PD, while NOS-based matrix showed AUC of 0.761. The RK-weighted connectome matrix (best among DKI) was significantly superior to the NOS-based connectome matrix. This means that parameter-weighted connectome matrix would be superior to conventional NOS-based matrix in diagnosing PD.

The RK-weighted connectome matrix (best among DKI) was also significantly superior to the FA-weighted (best among DTI) connectome matrices. This finding matches a previous study that reported that parameters derived from DKI analyses were significantly better than those from DTI analyses in diagnosing PD [35]. NODDI parameters represent the density and direction of neurites (axons and dendrites), and they are more specific markers of brain tissue microstructure [6]. There is a previous study which reported that both the DKI and NODDI parameters were useful for diagnosing PD [36]. However, the DKI parameter-weighted matrix performed significantly better than the NODDI parameter-weighted matrix in our study. The difference might have come from the difference in the evaluating methods; in that previous study regions of interest were placed on gray matter in measuring these values. In our study, the trained CNN based on RK-weighted matrix took into consideration many of the neural circuits comprehensively, which was indicated by the Grad-CAM result. According to Braak et al., as the disease progresses from stage 1 to stage 6 in PD patients, the deposition of Lewy bodies is thought to be spread to the upper brainstem and forebrain [37]. Because the PD patients included in this study had relatively long clinical course, we assume that such pathological conditions were present widely in the brain in many PD patients. DKI model, which quantifies non-Gaussian water diffusion [5], might be better to capture such pathological changes of PD than NODDI model when parameters derived from these models are used to weigh connectome matrix.

Little is known regarding *g*-ratio analysis for diagnosing PD. With *g*-ratio analysis, AVF, myelin volume fraction, and the ratio of the inner to outer myelinated axon diameter (*g*-ratio) can be derived [7]. AVF is an indicator of altered axons like ICVF in NODDI model [6, 7]. In this study, only ICVF and AVF showed performance with AUC of over 0.80 among the NODDI and *g*-ratio analysis, respectively. This does not conflict with a previous study [25], which reported reduction of ICVF in white matter indicating axonal degeneration in PD.

Traditionally, PD is characterized by degeneration of mesencephalic dopamine neurons. However, it has become known that several other brain regions and connections are known to be affected in PD [3]. A connection between basal ganglia and cerebellum is one of these connections in PD patients [38]. In previous studies, by using functional MRI in patients with PD, alterations in connectivity strength between basal ganglia and cerebellum have been suggested [39, 40]. In the current study, we found that neural connections between the basal ganglia and the cerebellum were focused on by ICVF and AVF-weighted connectome matrices with the Grad-CAM technique. The alterations of axons along those neural circuits might possibly be associated with alterations of function.

In this study, we customized the structure of the CNN, because we thought that the connectome matrix is different from general images in that distances between elements of matrices do not represent spatial distances. We used a filter size of 1 in all the convolutional layers. In addition, we avoided implementing max-pooling layers, which extract max values within *H* × *H* pixels (H = 2 or more). The customized CNN was found to be applicable to the imaging diagnosis with connectome matrices. While Grad-CAM result images are blurred generally [24], the customized structure of the CNN enabled pin-point visualization in Grad-CAM as shown in Fig. 4. The developed method would have a potential to identify neural circuit disorders in other neurological and psychiatric diseases.

This study has some limitations. First, although the Grad-CAM results indicated abnormalities in some of the neural circuits, the abnormalities were not proven histopathologically. Future analyses are required to consolidate the Grad-CAM findings obtained in this study. Second, because data from a large number of patients with PD were required to manage the overfitting problem associated with deep learning, patients with PD with variable disease durations (mean disease duration was over 10 years) were included in this study. Therefore, early diagnosis of PD is not possible with our models. It would also be possible that midbrain atrophy, which can be seen in some late-stage cases of PD, might have affected the performance of the model. Third, the model’s performance was not externally validated. Our study indicates that once the model is developed, it can be used to diagnose PD in the same institution. However, the model’s performance may not be as high as we reported when the method is applied to data obtained in other institutions. Fourth, connectome matrix can be constructed by using other data, such as functional MRI. However, we focused on analyzing connectome matrices based on diffusion MRI in this study. Application of this technique to connectome matrix based on functional MRI of PD patients’ needs to be investigated in the future. And finally, because we aimed to investigate the abnormalities in the neural circuits of PD compared with healthy controls, other diseases that show parkinsonism were not included in this study. Therefore, differentiation of PD from such diseases is not possible with our models. Diseases which show parkinsonism still needs to be differentiated by integrating clinical information and some test findings [41].

In conclusion, PD can be diagnosed by applying deep learning to DKI-, NODDI-, and *g*-ratio-weighted connectome matrices with moderate performance. Deep learning models trained with the DKI-weighted connectome matrix performed significantly better than those trained with the NOS-based connectome matrix. Alterations in neural connections between the basal ganglia on one side and the cerebellum on the contralateral side in PD patients were indicated by applying Grad-CAM technique to *g*-ratio-weighted and NODDI-weighted matrices.

## References

[1] Feigin VL, Abajobir AA, Abate KH, Abd-Allah F, Abdulle AM, Abera SF, Abyu GY, Ahmed MB, Aichour AN, Aichour I, Aichour MTE, Akinyemi RO, Alabed S, al-Raddadi R, Alvis-Guzman N, Amare AT, Ansari H, Anwari P, Ärnlöv J, Asayesh H, Asgedom SW, Atey TM, Avila-Burgos L, Frinel E, Avokpaho GA, Azarpazhooh MR, Barac A, Barboza M, Barker-Collo SL, Bärnighausen T, Bedi N, Beghi E, Bennett DA, Bensenor IM, Berhane A, Betsu BD, Bhaumik S, Birlik SM, Biryukov S, Boneya DJ, Bulto LNB, Carabin H, Casey D, Castañeda-Orjuela CA, Catalá-López F, Chen H, Chitheer AA, Chowdhury R, Christensen H, Dandona L, Dandona R, de Veber GA, Dharmaratne SD, Do HP, Dokova K, Dorsey ER, Ellenbogen RG, Eskandarieh S, Farvid MS, Fereshtehnejad SM, Fischer F, Foreman KJ, Geleijnse JM, Gillum RF, Giussani G, Goldberg EM, Gona PN, Goulart AC, Gugnani HC, Gupta R, Hachinski V, Gupta R, Hamadeh RR, Hambisa M, Hankey GJ, Hareri HA, Havmoeller R, Hay SI, Heydarpour P, Hotez PJ, Jakovljevic M(M)B, Javanbakht M, Jeemon P, Jonas JB, Kalkonde Y, Kandel A, Karch A, Kasaeian A, Kastor A, Keiyoro PN, Khader YS, Khalil IA, Khan EA, Khang YH, Tawfih A, Khoja A, Khubchandani J, Kulkarni C, Kim D, Kim YJ, Kivimaki M, Kokubo Y, Kosen S, Kravchenko M, Krishnamurthi RV, Defo BK, Kumar GA, Kumar R, Kyu HH, Larsson A, Lavados PM, Li Y, Liang X, Liben ML, Lo WD, Logroscino G, Lotufo PA, Loy CT, Mackay MT, el Razek HMA, el Razek MMA, Majeed A, Malekzadeh R, Manhertz T, Mantovani LG, Massano J, Mazidi M, McAlinden C, Mehata S, Mehndiratta MM, Memish ZA, Mendoza W, Mengistie MA, Mensah GA, Meretoja A, Mezgebe HB, Miller TR, Mishra SR, Ibrahim NM, Mohammadi A, Mohammed KE, Mohammed S, Mokdad AH, Moradi-Lakeh M, Velasquez IM, Musa KI, Naghavi M, Ngunjiri JW, Nguyen CT, Nguyen G, le Nguyen Q, Nguyen TH, Nichols E, Ningrum DNA, Nong VM, Norrving B, Noubiap JJN, Ogbo FA, Owolabi MO, Pandian JD, Parmar PG, Pereira DM, Petzold M, Phillips MR, Piradov MA, Poulton RG, Pourmalek F, Qorbani M, Rafay A, Rahman M, Rahman MHU, Rai RK, Rajsic S, Ranta A, Rawaf S, Renzaho AMN, Rezai MS, Roth GA, Roshandel G, Rubagotti E, Sachdev P, Safiri S, Sahathevan R, Sahraian MA, Samy AM, Santalucia P, Santos IS, Sartorius B, Satpathy M, Sawhney M, Saylan MI, Sepanlou SG, Shaikh MA, Shakir R, Shamsizadeh M, Sheth KN, Shigematsu M, Shoman H, Silva DAS, Smith M, Sobngwi E, Sposato LA, Stanaway JD, Stein DJ, Steiner TJ, Stovner LJ, Abdulkader RS, EI Szoeke C, Tabarés-Seisdedos R, Tanne D, Theadom AM, Thrift AG, Tirschwell DL, Topor-Madry R, Tran BX, Truelsen T, Tuem KB, Ukwaja KN, Uthman OA, Varakin YY, Vasankari T, Venketasubramanian N, Vlassov VV, Wadilo F, Wakayo T, Wallin MT, Weiderpass E, Westerman R, Wijeratne T, Wiysonge CS, Woldu MA, Wolfe CDA, Xavier D, Xu G, Yano Y, Yimam HH, Yonemoto N, Yu C, Zaidi Z, el Sayed Zaki M, Zunt JR, Murray CJL, Vos T (2017). Global, regional, and national burden of neurological disorders during 1990-2015: a systematic analysis for the Global Burden of Disease Study 2015. Lancet Neurol.

[2] Dorsey ER, Elbaz A, Nichols E, Abd-Allah F, Abdelalim A, Adsuar JC, Ansha MG, Brayne C, Choi JYJ, Collado-Mateo D, Dahodwala N, Do HP, Edessa D, Endres M, Fereshtehnejad SM, Foreman KJ, Gankpe FG, Gupta R, Hankey GJ, Hay SI, Hegazy MI, Hibstu DT, Kasaeian A, Khader Y, Khalil I, Khang YH, Kim YJ, Kokubo Y, Logroscino G, Massano J, Mohamed Ibrahim N, Mohammed MA, Mohammadi A, Moradi-Lakeh M, Naghavi M, Nguyen BT, Nirayo YL, Ogbo FA, Owolabi MO, Pereira DM, Postma MJ, Qorbani M, Rahman MA, Roba KT, Safari H, Safiri S, Satpathy M, Sawhney M, Shafieesabet A, Shiferaw MS, Smith M, Szoeke CEI, Tabarés-Seisdedos R, Truong NT, Ukwaja KN, Venketasubramanian N, Villafaina S, Weldegwergs K, Westerman R, Wijeratne T, Winkler AS, Xuan BT, Yonemoto N, Feigin VL, Vos T, Murray CJL (2018). Global, regional, and national burden of Parkinson’s disease, 1990-2016: a systematic analysis for the Global Burden of Disease Study 2016. Lancet Neurol.

[3] Andica C, Kamagata K, Hatano T, Saito Y, Ogaki K, Hattori N, Aoki S (2020). MR biomarkers of degenerative brain disorders derived from diffusion imaging. J Magn Reson Imaging.

[4] Basser PJ, Mattiello J, LeBihan D (1994). Estimation of the effective self-diffusion tensor from the NMR spin echo. J Magn Reson B.

[5] Jensen JH, Helpern JA, Ramani A, Lu H, Kaczynski K (2005). Diffusional kurtosis imaging: the quantification of non-Gaussian water diffusion by means of magnetic resonance imaging. Magn Reson Med.

[6] Zhang H, Schneider T, Wheeler-Kingshott CA, Alexander DC (2012). NODDI: practical in vivo neurite orientation dispersion and density imaging of the human brain. Neuroimage.

[7] Stikov N, Campbell JS, Stroh T (2015). In vivo histology of the myelin g-ratio with magnetic resonance imaging. Neuroimage.

[8] Dean DC, Sojkova J, Hurley S (2016). Alterations of myelin content in Parkinson’s disease: a cross-sectional neuroimaging study. PLoS One.

[9] Fornito A, Zalesky A, Breakspear M (2015). The connectomics of brain disorders. Nat Rev Neurosci.

[10] Fornito A, Zalesky A, Pantelis C, Bullmore ET (2012). Schizophrenia, neuroimaging and connectomics. Neuroimage.

[11] Kamagata K, Zalesky A, Hatano T, di Biase MA, el Samad O, Saiki S, Shimoji K, Kumamaru KK, Kamiya K, Hori M, Hattori N, Aoki S, Pantelis C (2018). Connectome analysis with diffusion MRI in idiopathic Parkinson’s disease: evaluation using multi-shell, multi-tissue, constrained spherical deconvolution. Neuroimage Clin.

[12] Kamagata K, Zalesky A, Yokoyama K, Andica C, Hagiwara A, Shimoji K, Kumamaru KK, Takemura MY, Hoshino Y, Kamiya K, Hori M, Pantelis C, Hattori N, Aoki S (2019). MR g-ratio-weighted connectome analysis in patients with multiple sclerosis. Sci Rep.

[13] LeCun Y, Bengio Y, Hinton G (2015). Deep learning. Nature.

[14] Krizhevsky A, Sutskever I, Hinton G (2012) ImageNet classification with deep convolutional neural networks. Advances in Neural Information Processing System 25 (NIPS 2012). Available via https://papers.nips.cc/paper/4824-imagenet-classification-with-deep-convolutional-neural-networks. Accessed 1 Sept, 2019.

[15] Yasaka K, Abe O (2018). Deep learning and artificial intelligence in radiology: current applications and future directions. PLoS Med.

[16] Chartrand G, Cheng PM, Vorontsov E, Drozdzal M, Turcotte S, Pal CJ, Kadoury S, Tang A (2017). Deep learning: a primer for radiologists. Radiographics.

[17] Yasaka K, Akai H, Kunimatsu A, Kiryu S, Abe O (2018). Deep learning with convolutional neural network in radiology. Jpn J Radiol.

[18] Lakhani P, Sundaram (2017). Deep learning at chest radiography: automated classification of pulmonary tuberculosis by using convolutional neural networks. Radiology.

[19] Larson DB, Chen MC, Lungren MP, Halabi SS, Stence NV, Langlotz CP (2018). Performance of a deep-learning neural network model in assessing skeletal maturity on pediatric hand radiographs. Radiology.

[20] Yasaka K, Akai H, Abe O, Kiryu S (2018). Deep learning with convolutional neural network for differentiation of liver masses at dynamic contrast-enhanced CT: a preliminary study. Radiology.

[21] Prevedello LM, Erdal BS, Ryu JL, Little KJ, Demirer M, Qian S, White RD (2017). Automated critical test findings identification and online notification system using artificial intelligence in imaging. Radiology.

[22] Kiryu S, Yasaka K, Akai H, Nakata Y, Sugomori Y, Hara S, Seo M, Abe O, Ohtomo K (2019). Deep learning to differentiate parkinsonian disorders separately using single midsagittal MR imaging: a proof of concept study. Eur Radiol.

[23] Yasaka K, Akai H, Kunimatsu A, Abe O, Kiryu S (2018). Liver fibrosis: deep convolutional neural network for staging by using gadoxetic acid-enhanced hepatobiliary phase MR images. Radiology.

[24] Selvaraju RR, Cogswell M, Das A, Vendatam R, Parikh D, Batra D (2016) Grad-CAM: visual explanations from deep networks via gradient-based localization. Available via https://arxiv.org/abs/1610.02391. Accessed 1 Oct 2020

[25] Andica C, Kamagata K, Hatano T, Saito Y, Uchida W, Ogawa T, Takeshige-Amano H, Hagiwara A, Murata S, Oyama G, Shimo Y, Umemura A, Akashi T, Wada A, Kumamaru KK, Hori M, Hattori N, Aoki S (2020) Neurocognitive and psychiatric disorders-related axonal degeneration in Parkinson's disease. J Neurosci Res 98:936-949 10.1002/jnr.24584 PMC7154645 32026517

[26] Andica C, Kamagata K, Hatano T, Saito A, Uchida W, Ogawa T, Takeshige-Amano H, Zalesky A, Wada A, Suzuki M, Hagiwara A, Irie R, Hori M, Kumamaru KK, Oyama G, Shimo Y, Umemura A, Pantelis C, Hattori N, Aoki S (2019) Free-Water Imaging in White and Gray Matter in Parkinson's Disease Cells 8:839 10.3390/cells8080839 PMC6721691 31387313

[27] Postuma RB, Berg D, Stern M, Poewe W, Olanow CW, Oertel W, Obeso J, Marek K, Litvan I, Lang AE, Halliday G, Goetz CG, Gasser T, Dubois B, Chan P, Bloem BR, Adler CH, Deuschl G (2015). MDS clinical diagnostic criteria for Parkinson’s disease. Mov Disord.

[28] Andersson JLR, Sotiropoulos SN (2016). An integrated approach to correction for off-resonance effects and subject movement in diffusion MR imaging. Neuroimage.

[29] Tabesh A, Jensen JH, Ardekani BA, Helpern JA (2011). Estimation of tensors and tensor-derived measures in diffusional kurtosis imaging. Magn Reson Med.

[30] Daducci A, Canales-Rodriguez EJ, Zhang H, Dyrby TB, Alexander DC, Thiran JP (2015). Accelerated Microstructure Imaging via Convex Optimization (AMICO) from diffusion MRI data. Neuroimage.

[31] Mohammadi S, Carey D, Dick F (2015). Whole-brain in-vivo measurements of the axonal G-ratio in a group of 37 healthy volunteers. Front Neurosci.

[32] Greve DN, Fischl B (2009). Accurate and robust brain image alignment using boundary-based registration. Neuroimage.

[33] Desikan RS, Segonne F, Fischl B (2006). An automated labeling system for subdividing the human cerebral cortex on MRI scans into gyral based regions of interest. Neuroimage.

[34] Duchi J, Hazan E, Singer Y (2011). Adaptive subgradient methods for online learning and stochastic optimization. J Mach Learn Res.

[35] Kamagata K, Tomiyama H, Hatano T, Motoi Y, Abe O, Shimoji K, Kamiya K, Suzuki M, Hori M, Yoshida M, Hattori N, Aoki S (2014). A preliminary diffusional kurtosis imaging study of Parkinson disease: comparison with conventional diffusion tensor imaging. Neuroradiology.

[36] Kamagata K, Zalesky A, Hatano T, Ueda R, di Biase MA, Okuzumi A, Shimoji K, Hori M, Caeyenberghs K, Pantelis C, Hattori N, Aoki S (2017). Gray matter abnormalities in idiopathic Parkinson’s disease: evaluation by diffusional kurtosis imaging and Neurite orientation dispersion and density imaging. Hum Brain Mapp.

[37] Braak H, Ghebremedhin E, Rüb U, Bratzke H, Del Tredici K (2004). Stages in the development of Parkinson’s disease-related pathology. Cell Tissue Res.

[38] Milardi D, QUartarone A, Bramanti A (2019). The cortico-basal ganglia-cerebellar network: past, present and future perspectives. Front Syst Neurosci.

[39] Kawabata K, Watanabe H, Bagarinao E, Ohdake R, Hara K, Ogura A, Masuda M, Kato T, Tsuboi T, Maesawa S, Katsuno M, Sobue G (2020). Cerebello-basal ganglia connectivity fingerprints related to motor/ cognitive performance in Parkinson’s disease. Parkinsonism Relat Disord.

[40] Husárová I, Lungu OV, MareLcek R (2014). Functional imaging of the cerebellum and basal ganglia during predictive motor timing in early Parkinson’s disease. J Neuroimaging.

[41] Tolosa E, Wenning G, Poewe W (2006). The diagnosis of Parkinson’s disease. Lancet Neurol.

